# The impact of three types of writing intervention on students’ writing quality

**DOI:** 10.1371/journal.pone.0218099

**Published:** 2019-07-18

**Authors:** Pedro Rosário, Julia Högemann, José Carlos Núñez, Guillermo Vallejo, Jennifer Cunha, Celestino Rodríguez, Sonia Fuentes

**Affiliations:** 1 Department of Applied Psychology, Universidade do Minho, Braga, Portugal; 2 José Carlos Núñez, Department of Psychology, Universidad de Oviedo, Oviedo, Spain; 3 Facultad de Educación, Universidad Central de Chile, Santiago de Chile, Chile; Kyoto University, JAPAN

## Abstract

Students’ writing constitutes a topic of major concern due to its importance in school and in daily life. To mitigate students’ writing problems, school-based interventions have been implemented in the past, but there is still a need to examine the effectiveness of different types of writing interventions that use robust design methodologies. Hence, the present study followed a longitudinal cluster-randomized controlled design using a multilevel modeling analysis with 370 fourth-grade students (nested in 20 classes). The classes were randomly assigned to four conditions: one comparison group and three writing types of writing interventions (i.e., week-journals, Self-Regulation Strategy Development (SRSD) instruction and SRSD plus Self-Regulated Learning (SRL) program using a story-tool), with five classes participating in each condition. Data supports our hypothesis by showing differences between the treatment groups in students’ writing quality over time. Globally, the improvement of students’ writing quality throughout time is related to the level of specialization of the writing interventions implemented. This is an important finding with strong implications for educational practice. Week-journals and writing activities can be easily implemented in classrooms and provides an opportunity to promote students’ writing quality. Still, students who participated in the instructional programs (i.e., SRSD and SRSD plus story-tool) exhibited higher writing quality than the students who wrote week-journals. Current data did not find statistical significant differences between results from the two instructional writing tools.

## Introduction

In the last decades, students’ writing problems throughout schooling have been discussed as a topic of educational concern due to the importance of writing in school and life success (e.g., employment) (e.g., [1–2]). To mitigate students’ writing problems, curriculum reforms have been implemented in different educational systems, and researchers have been investigating the efficacy of school-based interventions in improving students’ writing (e.g., free writing activities, strategy instruction as Self-Regulation Strategy Development, SRSD) (e.g., [3–6]). Still, there is a need to disclose evidence on the effectiveness of different types of writing interventions using robust design methodologies. Data is expected to help researchers, school administrators and teachers organize school-based interventions and promote students’ writing skills [7].

To analyze the effectiveness of three writing interventions (i.e., week-journals, SRSD, and SRSD plus a Self-Regulated Learning program using a story-tool) on fourth graders motivational variables and writing quality a cluster-randomized controlled design was conducted for twelve weeks.

### Promoting students’ writing performance

Previous research has strengthened the idea that writing is one of the most powerful and fundamental tools, not only to learn, but to communicate and share knowledge [8–9]. In fact, the ability to communicate and express one’s thoughts and ideas through writing is truly essential for success at school and in further education [10]. This section provides an overview of three types of writing interventions examined in the current study.

#### Writing week-journals

Students’ motivation and engagement in writing are likely to grow in learning environments providing many opportunities and encouragements for students to express themselves through writing [11–14]. Journal writing is a practice that can be easily implemented in classrooms without much effort, time, or resources (e.g., [5,15]). Journals are a type of free writing that is informal and personal [16–17] and have gained popularity among the activities aiming at promoting writing [17] and students’ confidence in writing [18]. The nature of this educational tool allows students to write freely without strict directions, restrictions or assessment purposes [16]. While writing journals, students will choose their writing topic [19], engage deeply in their writing activities [17] and improve their writing skills and creativity [16]. Furthermore, writing journals allow students to enhance their reflection skills, critical thinking, self-expression, self-regulated skills, and knowledge [17].

Notwithstanding the potential positive influence of writing journals on students’ motivation and writing performance (e.g., [16,18]), findings from the extant research are not consistent. Prior research (e.g., [4,16,20–21]) found no statistical evidence on the effectiveness of free writing on students’ writing quality. But, a recent study with fourth graders concluded that students who wrote weekly journals for twelve weeks showed a higher improvement on the quality of their compositions, than that achieved by students in the comparison group [15]. Despite these encouraging findings, students in the experimental group reached a plateau after the first three weeks of writing journals, which might indicate that this type of intervention may not be sufficient to foster progress on writing quality.

#### Writing and self-regulation

Considerable progress has been made in the last thirty-five years to understand the role of self-regulation in writing. Not surprisingly, research found that skilled writers master self-regulated learning competencies (e.g., self-set goals, self-reinforcement) [22], and also that many students struggle with writing [23]. This may happen because effective writing requires: (i) high levels of self-regulation and attentional control to manage the writing environment; (ii) knowledge of the writing topic, genre, processes and skills involved in writing [22]; (iii) strategies for planning, text production [24–25] and monitor the writing activity [26] to meet specific self-set goals [27].

Three decades ago, Karen Harris and Steve Graham built the Self-Regulation Strategy Development model (i.e., SRSD model; [28])—an instructional program designed to enhance writing and self-regulation strategies. SRSD was designed to attain the three major goals, as follows [29]: (i) to help students develop the knowledge and skills needed to manage the writing strategies involved in the writing processes (i.e., planning, writing, revising and editing); (ii) to support students using the strategies and self-regulatory skills (e.g., goal-setting, self-instruction, self-assessment, self-reinforcement) while monitoring and managing their own writing (e.g., [30–32]); and finally (iii) to help students develop positive attitudes and beliefs about themselves as writers [31,33–34]. In fact, when students perceive themselves as self-efficacious in writing, they are likely to exhibit good writing quality and invest effort while carrying out a writing task [34–36].

The meta-analysis by Graham et al. [5] analyzed the impact of the SRSD model on students’ writing and found that adding self-regulation instruction (e.g., goal setting and self-assessment) to strategy instruction can improve the overall writing quality of typical developing writers and, in most cases, of struggling writers. The benefits of participating in SRSD programs are well established in literature (e.g., [23]), but further research is needed to explore complementary forms infused in regular curriculum that may increase the teaching of writing strategies [3,5,15]. Recently Rosário and colleagues (e.g., [15,37–41]) discussed the use of story-tools in class as a successful strategy to foster students’ motivation, and promote self-regulated learning (SRL). Based on the extant evidence which supports the role of stories to promote SRL, current authors believe that infusing story-tools in the regular curriculum combined with writing instruction (i.e., SRSD) may be beneficial for increasing the levels of writing quality.

#### Story-tools to promote SRL

Stories, traditional tales and fables are well-known ways of delivering knowledge [40], to promote children’s development [42–45], imagination [46], and self-reflection about their own behaviors [40]. Bearing this in mind, researchers in Iberian Peninsula, created SRL story-tools programs that focus on promoting SRL through different types of narratives. The *Yellow trials and tribulations* [45] is a story–tool developed to promote SRL at elementary school, and was used in the present study. This narrative tells the story of the disappearance of the color Yellow from the Rainbow and describes the adventures experienced by Yellows’ friends, the other colors of the rainbow, whilst searching for Yellow. Along this quest in search for Yellow, who should not be left alone, the other colors of the rainbow met new friends and learned various useful SRL strategies to overcome the obstacles found along the way.

This story-tool was designed to promote students’ SRL strategies (e.g., goal-setting, self-reflection, strategic planning, and organizational strategies), to increase motivation and academic achievement [47]. This tool is grounded on the social cognitive framework [48], and assumes that contextual variables and learning settings play important roles in students’ motivation and self-regulation [47]. The stories in each chapter of the story-tool address the PLEE cyclical model: Planning, Execution and Evaluation (see [40] for a more detailed explanation), which is rooted in the SRL model by Zimmerman [49–50]. Students are expected to regulate their school behaviors in three cyclical phases: forethought (i.e., processes prior to learning), performance control (i.e., processes while learning), and self-reflection (i.e., processes after learning). The former model presents a recursive structure, through two paths of logic. The process is derived from Planning through Execution to Evaluation, but the same cyclical nature is also reset in each phase, thus reinforcing the self-regulation logic of the process. These two structuring loops, throughout and within the phases, reinforce the SRL synergy strengthening the process [38,40,51].

Modeling and teaching the learning strategies (e.g., goal-setting, strategic planning, organizational strategies), embedded in the story-tool underlies on three types of knowledge [38]: (i) the declarative knowledge—learning the meaning of a learning strategy (e.g. know what taking notes is); (ii) the procedural knowledge, that is related to learn how to implement these learning strategies (e.g., know how to take notes in class); and, finally, (iii) the conditional knowledge that demands students to know when it is more appropriate to use a specific learning strategy in a particular learning context (e.g., when it is more useful to take notes) [52]. For example, in chapter 6 of the story-tool [45], the Ant General, one of the characters, explained the planning phase to his *troops* (i.e., declarative knowledge): “in order to plan, we have to decide what we need to know and what we need to do for everything to run smoothly. Afterwards, to avoid any problems, we allocate time for each task” (p. 27).

Each chapter provides students with the opportunity to acquire, practice and reflect on the use of the SRL strategies embedded in each phase of the PLEE model. This tool allows the analysis of the characters’ behavior which are similar to those of children in real life situations (e.g., *the Bird-Teacher told the little birds a story about a lazy deer who did not listened to the teacher advice’s friends and hurt himself while competing with a grasshopper*), hence helping students to reflect on what they may learn with the characters’ behaviors. This experiential closeness fosters children’s engagement in learning [40]. For example, it is expected for students to transfer the content learned throughout the story to the process of writing compositions.

### Present study

Driven by the worldwide need to promote students’ writing quality and to examine the impact of various types of writing interventions tailored to students’ needs and school resources, the current study examines the impact of three types of writing interventions (i.e., week-journals, SRSD, and SRSD plus a SRL program using a story-tool) on students’ writing quality.

Research data on the positive effects of using week-journals to improve students writing quality is inconsistent; however recent data from a controlled study [15] reported that students using week-journals improved the quality of writing after the first three weeks, and then reached g a plateau on the following weeks. These findings suggest that this tool solely may not be sufficient to sustain students’ progress on the writing quality. Moreover, the corpus of research on SRSD is vast and data has consistently indicated the efficacy of the SRSD programs to improve the quality of writing [5,23]. Finally, Rosário and colleagues have been advocating for the last decade the merits of using story-tools to promote SRL [40,53]. The current research aims to examine the potential positive effects of adding a story-tool to SRSD program. This design addresses the call by authors [3,5] to explore ways of promoting the teaching of writing strategies embedded in regular curriculum. Children read and learn stories in class and at home; in fact, stories make up part of their lives and play a vital role in their growth and development. While reading books and reflecting on the messages conveyed, children are expected to learn how to think, and also to learn about everyday tasks [42, 43]. For these reasons, we believe that adding a story tool to the training of writing strategies is likely to improve children writing quality. Findings are expected to add literature on writing quality and improve educators’ practices on writing.

In addition, the impact of several potentially moderating variables, such as self-regulation in writing, self-efficacy in writing, attitude towards writing, prior achievement in writing, gender, age and interactions between these variables and will be examined. Based on extant literature (e.g., [15,30,39]) we hypothesize that: (i) students’ writing quality of the three intervention groups will be higher when compared to students in the comparison group; (ii) students’ writing quality in the SRSD and SRSD plus the SRL story-tool conditions will be higher when compared to students in the week-journal condition; (iii) all covariates will be significantly related with students’ writing quality. No hypothesis will be made regarding the conditions SRSD and SRSD and the SRL story-tool because literature lacks data in this regard. This step of the research is exploratory.

## Method

### Design and participants

#### Design

**Fig 1:**
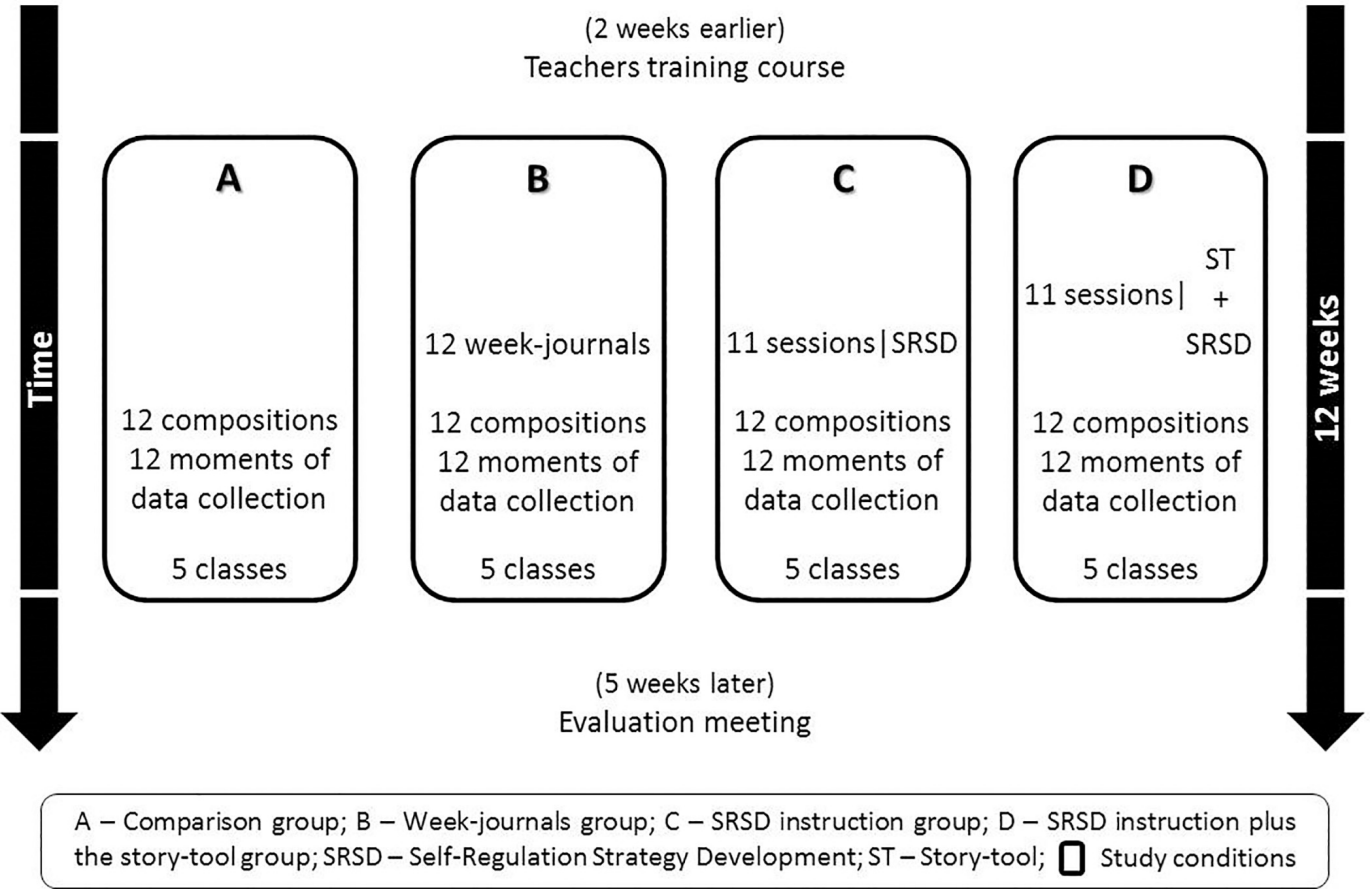
Schematic of each treatment condition and procedures.

The present study was conducted with fourth grade students, the final grade level in Portuguese elementary school. The Portuguese Ministry of Education approved the study by giving their written consent (n. 036000004). This study was reviewed and approved by the ethics committee of the Universidade do Minho. The study followed a longitudinal cluster-randomized controlled design for twelve weeks, in 18 public schools in the north of Portugal. The participating teachers and their fourth-grade students were randomly assigned to the four conditions, with five classes participating in each condition (i.e., Groups A, B, C and D; see Fig 1). This methodology is useful to access the comparative effectiveness of experimental conditions that vary in their practices. Moreover, this tool helps avoid “contamination” between those participants receiving the intervention and those who are not, preventing that the treatment effect would be compromised [54]. During the twelve weeks of the study, students on the comparison condition (Group A) did not participate in any type of program focused on writing instruction. Teachers were instructed to follow the regular Portuguese writing curriculum to meet fourth grade level teaching requirements. According to the Directorate-General for Education and the Minister of Education and Science [55] this included teaching students about grammar, vocabulary, spelling, sentence construction, punctuation, handwriting, organization and revision of different types of text (i.e., narrative, informative, descriptive, letters, invitations, and texts using direct speech). In group B students wrote a journal on a weekly basis for 12 weeks. Students in group C and D were given writing instructions following the SRSD model; in group D the story-tool “*Yellow Trials and Tribulations*” [45] were added to the treatment received by the group C (see Fig 1).

#### Participating students and their teachers

The participants were 370 (183 girls) fourth graders nested in 20 classes from 18 public elementary schools in the north of Portugal. All the participants had Portuguese as their home language, aged between 9 and 10 (*M* = 9.45, *SD* = .51). The fourth-grade classes were randomly assigned to four groups: A (*N* = 92); B (*N* = 90); C (*N = 98*); and D (*N = 90*). Students with special education needs (i.e., specific learning disorder and learning disabilities) were excluded from the data analyses.

All the 20 teachers, 17 were female, aged between 34–56 years (*M* = 42.4, *SD* = 6.59) had an undergraduate degree and experience in teaching ranging between 12 and 34 years (*M* = 21.5, *SD* = 6.16). Class sizes ranged between 10 and 23 (*M* = 20.38, *SD* = 4.75). None of the teachers enrolled in the study reported having received specific writing instruction in their professional development.

After receiving the consent from the Portuguese Ministry of Education, an email explaining the overall study objectives was sent to 26 public schools located in northern part of Portugal. Eighteen schools (a response rate of 69.2%) and 20 teachers agreed to participate in our research. In these schools, the families were lower-middle classes, as noted by the high percentage of students (40%) receiving free or reduced-price lunches. These demographics were collected from the offices of the participating schools. A letter informing about the study was sent out to ask permission for the children participate the study. Participants’ confidentiality was assured (e.g., eliminating the names and researchers’ personal notes that could link the participants to their teachers or schools). All students returned the signed parental consent forms. Finally, the 20 teachers (classes) who agreed to participate were randomly assigned to the four treatment conditions (i.e., comparison group and three experimental groups). Teachers were blind to the purpose of the study and all agreed to follow the fourth grade Portuguese curriculum (e.g., variety of text genres, grammar and punctuation) throughout the study.

### Training

Two weeks prior to the beginning of the study, a training course with two modules was delivered separately to all participating teachers within the same condition (i.e., Groups A, B, C and D). The first module (9 h) presented and discussed of the general framework (e.g., genre of the compositions, protocol of the weekly administration of the questionnaires by the research team) and the assessment measures (e.g., rating scale for teachers to assess the quality of the compositions). Participants were informed that following the protocol was a requirement to participate, and all agreed.

In the second module (8h) teachers worked collaboratively with researchers and assistant researchers in 2-hour sessions over a span of four days (i.e., 20 pre-service teachers) on the assessment of the overall quality of the children compositions. The training on how to use the rating scale (see measures) followed a hands-on approach. Teachers selected a set of compositions made by their students in the third grade, and switched those compositions with their colleagues and assistant researchers on a random basis. Each composition was assessed independently using the rating scale. After scoring each composition, teachers and research assistants met and discussed scores to reach a consensus. To ensure reliability of the assessment process, each teacher assessed eight compositions over the four days, each time with a different research assistant. Kappa value was calculated using the Coder Comparison Queries in the Navigation View of the NVivo software. In the end of the training the Kappa value of the 20 dyads ranged between .80 and .86 (*M =* .82) which can be labeled as “almost perfect” according to Landis and Koch [56].

Five weeks post-intervention, all teachers from the four groups participated in a three-hour evaluation meeting to analyze their experiences during the intervention (e.g., comments and suggestions that could help in future research), and discuss preliminary data (see, [57–58]) from the standardized exam in Portuguese language. In this meeting, teachers from the four groups declared, as agreed, to have followed the national writing curriculum (e.g., teaching grammar, punctuation and the other types of genres) to meet fourth grade level expectations. Teachers who fully participated in the research were offered a 27-hour (1 ECTS) training course about the learning and instruction processes.

### Treatment integrity

To assure the integrity of the implementation of the protocol conducted by the teachers, four different measures were used: i) all teachers were delivered dossiers with session record sheets (see, [59]) including the elements and activities for each session. These dossiers helped teachers monitor the steps for each session. Each of the activities intended for the session and group were detailed in topics and teachers were asked to check it off when the activity was completed (e.g., teachers are expected to maintain a silent class while students are writing compositions; compositions are expected to be written in 45 minutes; journals are due to be kept in the classroom in a closet under the responsibility of a research assistant; students write about the composition topic assigned to that week topic; teachers do not make comments on students week-journal entry; teachers do not suggest topics for the week-journals); ii) Moreover, teachers were asked to write a short diary explaining how they followed protocol, and if not, to explain why; iii) Additionally, on a random basis, a research assistant observed 30% of the sessions using the same session record sheets. These research assistants also wrote a short diary describing teachers’ adherence to the protocol; iv) Finally, during the duration of the intervention, on a weekly basis, the principle investigator met with the researchers and research assistants and engaged in each condition separately. These meetings addressed project issues and adherence to protocol of each condition (e.g., analysis of record sheets data). Afterwards, research assistants enrolled in assessing compositions met with their *dyad teacher* and discussed the same issues. The major goal of these meetings was to prevent the teachers and the researcher (enrolled in delivering training lessons of conditions C and D) from withdrawing from the planned protocol by adding new components based on their experience of what was working.

Treatment fidelity was high for the writing composition sessions. Teachers reported adherence to the protocol was 95% (*SD* = 2.77, range 90–100). Data from the observations of both intervention sessions indicated that teachers completed 93% of the activities (*SD* = 3.24, range 85–98). Data from the teachers’ diaries and research assistants allowed to conclude that discrepancies in the assessment may be due to different interpretation of teachers’ behaviors in class (e.g., classroom management issues such as maintain complete silence in class while students were doing their compositions, and responding to students with “leading questions”).

But Concerning the treatment fidelity of the week-journal sessions, data indicated a good treatment receipt. Research assistants who enrolled in this treatment condition reported to have completed 87% of the tasks (*SD* = 2.62, range 81–90) across all sessions. Data from the observations of this intervention sessions indicated that research assistants completed 84% of the tasks (*SD* = 3.06, range 80–90).

Lessons for the groups C (*SRSD instruction)* and D (*SRSD instruction plus the story-tool)* were delivered by one of the authors of this paper with training in SRL and writing strategies. This researcher followed the treatment fidelity procedure previously described.

Treatment fidelity for lessons of conditions C and D was high for both. Researcher reported 88% (*SD* = 1.61, range 85–90) and 85% (*SD* = 3.62, range 79–90) of the activities completed across all lessons, respectively. Data from the observations of both conditions indicated that researcher completed 84% of the activities (*SD* = 1.94, range 81–87) and 82% (*SD* = 2.55, range 78–85), respectively.

### Specific intervention procedures for all participating students

For twelve weeks, on each Monday morning during regular Portuguese language class, all students’ from the four conditions wrote a composition in 45 minutes. The composition topic was sent by email to all teachers each Sunday evening (e.g., *Imagine that you were on a boat school trip*. *Suddenly*, *the boat was caught in a big storm and shipwrecked*. *Write a story about your adventure as a castaway and your life in a desert island)*. Along the duration of the investigation, students wrote one story each week. Compositions were assessed individually and every Thursday after school, along 12 weeks, the dyads (i.e., teacher and a randomly assigned research assistant) met to find consensus on the scores given. Finally, the graded compositions were delivered to students each Friday. Additionally, every Friday afternoon for approximately 25 minutes, all students from the four conditions were asked to fill in questionnaires to assess SRL strategies in writing, attitude towards writing and self-efficacy. The research assistants administrated these instruments in class.

#### Comparison group (group A) and Week-Journals (group B)

During the twelve weeks of the study, students on the comparison condition and weekly-journals did not participate in any type of writing instruction, besides the writing of the weekly compositions proposed for this research. Teachers were instructed just to follow the regular writing curriculum [55] to meet fourth grade level expectations.

Additionally, for twelve weeks, students in the week-journals condition (i.e., group B) wrote a journal in 25 minutes each Friday morning under the supervision of a research assistant. While students were writing their journals they did not receive any instructions, nor feedback afterwards. Prior to the beginning of the study, participants’ confidentiality was assured, by telling students that the journals would only be used for research purposes (i.e., teachers did not read the journals). Each student received a notebook “journal” to write their weekly entries (i.e., approximately ten lines) about their week’s events at school or at home. Journals were kept in the classroom in a closed box and were the responsibility of a research assistant.

#### General instructional procedures (intervention conditions C and D)

SRSD writing instruction, as well as the topics for condition D, were delivered along eleven sessions on a weekly basis, by one of the authors, during regular Portuguese language lessons. The length of the sessions for students in group C and D was 45 minutes. Both intervention conditions are briefly described in S1 Appendix. An extended description of the lessons and materials suggested for instruction is provided elsewhere [53].

#### SRSD instruction (intervention condition–group C)

The writing instruction followed the six stages of the SRSD model [25,28] as follows: (i) development of background knowledge; (ii) discussion and description of the strategies to be learned; (iii) modeling the use of those strategies; (iv) memorization of those strategies; (v) supporting of the strategies; and, finally, (vi) independent performance. In the present study, instruction started at the first stage and continued into the following stages (see S1 Appendix). Despite acknowledging the sequence of the content, we followed Harris and Graham [28] and asked students to memorize the mnemonics taught (strategy from stage four) since session 1. Thus, this *stage* was recalled at the beginning of every session to analyze if students had memorized the mnemonics [60]. A number of self-regulation procedures were also taught to students, including self-monitoring while planning their stories, self-reinforcement and self-assessment [60]. The materials for teaching writing narratives using the SRSD model were translated to Portuguese and used by fourth graders and teachers in class.

#### Writing strategies

In the first sessions, students learned a general strategy to apply while writing their compositions. This strategy included three steps, represented by the mnemonic POW: *Pick my ideas (i*.*e*., *decide what to write about)*, *Organize my notes (i*.*e*., *organize writing ideas into a writing plan)*, *Write and say more* (i.e., continue to modify, upgrading the plan while writing). For example, on the second step of POW (i.e., *organize my notes)* students were taught a genre-specific strategy for writing notes for each part of the story: the mnemonic S-A-C [principal steps of a story: Setting (*S*), action (*A*) and conclusion (*C*)] (see [53]). To help students become familiar with the S-A-C mnemonic, students were taught to ask themselves the following six questions, aligned with the three S-A-C steps: *Where does the story take place*? *When does the story take place*? *Who are the main characters (describe them)*? *What do the main characters do or want to do (sort them in the right way)*? *How does the story end*? *How do the main characters and the others feel*? For writing notes, students were presented with a graphic organizer (see [53]).

#### Strategy instruction

The strategy instruction followed the SRSD model [28], however the time spent on each stage was adjusted to the design of the current study. As shown in S1 Appendix, *lesson one and two* aimed to develop students’ prior knowledge on composition and to discuss and explore the characteristics of a good story. General writing strategies (i.e., POW) were presented and discussed with students. Students’ negative beliefs about writing performance were also discussed, and students were encouraged to transform negative thoughts into positive beliefs (e.g., "I can do it, if I use the right strategy”). In *lesson three and four*, students revisited the general writing strategies (i.e., POW) and discussed the SRL strategies (i.e., self-instructions, goal setting, self-assessment and self-reinforcement) they will use during and after writing a story. In *lesson five*, *six and seven* the planning, writing and assessing of compositions using general (i.e., POW) and SRL strategies (i.e., self-instructions, goal setting, self-assessment and self-reinforcement) were modeled collaboratively in class. Modeling the use of strategies helped students to learn to apply these strategies and to develop competencies, attitudes and beliefs, while writing independently. *Lesson eight*, *nine* and *ten* focused on strengthening students’ abilities for independent planning, writing and assessing of stories by using general (i.e., POW) and SRL strategies (i.e., self-instructions, goal setting, self-assessment and self-reinforcement). The work on these lessons aimed to wean students off the graphic organizer [60]. Finally, in *lesson eleven* students wrote, without support, a composition, using the strategies learned. Still, as suggested by authors [61], if any story elements were not included, the previous stages were recalled.

#### SRSD instruction plus the story-tool (intervention condition–group D)

In the current study, the *Yellow Trials and Tribulations* story-tool [45] was used to help students learn a set of learning strategies and apply them into the story-tool learning context while reflecting upon their own writing activities (i.e., on how and when to implement the general and SRL strategies). Sessions for the group D were preceded by the reading out loud of one or two chapters of the book in class. During the reading, small breaks were made and students were invited to discuss and analyze what was happening in the story plot (see [40,53]). During the session students did the same writing tasks as students in group C. The Appendix aligns the stages from SRSD (i.e., group C) with the chapters of the story-tool.

### Instruments and measures

#### Self-regulated learning strategies inventory (SR_W)

The SRL Strategies Inventory [38] assesses nine SRL strategies concerning the three phases of the SRL process (i.e., planning, execution and evaluation). In the preset study, this scale was adapted with the aim of assessing the SRL strategies used while writing: *Planning* (i.e., ‘‘I make a plan before I begin writing. I think about what I want to say and how I need to write it”), *Execution* (i.e., “While I write my composition I follow my plan”, and *Evaluation* (i.e., ‘‘I compare the grades I received with the goals I set for that subject.”). The 9-items were scored on a 5-point *Likert* scale, ranging from 1 (never) to 5 (always). Cronbach’s alpha in this study was .80. Data from the confirmatory factorial analysis run support the construct validity of this measure. The model fits well data [χ^2^ (25) = 53.639; p < .01; AGFI = .907; TLI = .900; CFI = .927; SRMR = .058; RMSEA = .076 (.048-.104)]. The factor weights of the nine items ranged from .507 to .703 (all statistically significant at *p* < .001). After fit the model, none of the modification indexes was greater than 5.00.

#### Attitude towards writing (AT_W)

Each of the nine items from the writing attitude survey [34] asked students to indicate how they felt when they engaged in writing activities at school or at home (e.g., *How do you feel when you think you have to write instead of being able to play*?*)*. Students were asked to mark one of the four images of Garfield the Cat on a 4-point *Likert* scale (1 = very unhappy; 4 = very happy). This scale was, in the present study, translated and adapted to the Portuguese population. Cronbach’s alpha in this study was .86. The construct validity analysis yielded data supporting a unifactorial model [χ^2^(25) = 34.086; *p* > .05; AGFI = .933; TLI = .976; CFI = .983; SRMR = .034; RMSEA = .043 (.000-.076]. The factorial weights of the nine items ranged from .660 to .750 (all statistically significant at p < .001). After fit the model, none of the modification indices was greater than 6.00. All data suggest construct validity.

### Self-efficacy in writing (SE_W)

Students’ self-efficacy for planning and writing a story was assessed with five-items [60]. An example of an item was *“When writing a paper*, *I have trouble finding the right words for what I want to say”*. The five-items were scored on a 4-point *Likert* scale (1 = strongly disagree; 4 = strongly agree). This scale was translated and adapted to the Portuguese population. Cronbach’s alpha in this study was .71. Data from the confirmatory factorial analysis run support the construct validity of this measure. [χ^2^(3) = 5.646; *p* > .05; AGFI = .943; TLI = .945; CFI = .983; SRMR = .026; RMSEA = .067 (.000-.151)]. The factorial weights of the five items are statistically significant at *p* < .001). After fit the model, the modification indices do not suggest any changes in the model.

### Writing performance

Individual notebooks were delivered for each participating student for research purposes. The notebooks had twelve parts (i.e., one for each of the twelve independent writing moments) and each had three subparts: (i) a lined page for the writing of the composition; (ii) a rating scale for students to review and self-assess the quality of their compositions; and finally, (iii) a checklist for the individual feedback given by the teacher.

#### Compositions

In order to assess the writing quality of students’ compositions, a holistic rating scale was used based on the criteria defined in the Educational Progress Test (i.e., a standardized exam) in Portuguese language for fourth graders [62]. The rating scale assesses topics such as (i) title; (ii) organization (introduction, main body paragraph, ending), (iii) grammatical correctness of sentences (e.g., active verbs, use of direct speech, descriptive adjectives, punctuation, morphology) (iv) coherence; (v) originality; (vi) sentence structure, (vii) word choice; (viii) spelling errors. Prior to scoring, all narratives were typed into a word document and the number of words were counted. Students’ personal information was removed and punctuation, spelling and capitalization were corrected to minimize bias that might influence the scoring process as suggested by the literature (e.g., [34]). Teachers were encouraged to read the composition to obtain a general impression of overall writing quality. Compositions were then scored on fourteen 5-point *Likert* scales (1 = low quality; 5 = high quality), ranging from 0 to 65 points. All compositions from the same class were scored independently by a dyad (teacher-research assistant) using the mentioned rating scale. Each dyad met every week to find a consensus about the grades for each composition as previously stated (see procedures subsection). Cohen’s Kappa coefficient showed an inter-rater agreement that ranged among the 20 dyads between .82 and .90 (*M =* .86, *SD* = .023) which can be labeled as “almost perfect” according to authors [63]. The compositions rated for each topic were assessed and the final score were delivered before students write the following composition.

#### Journals

Feedback on the week-journals was not provided to students. In the end of the study four new research assistants who were unfamiliar with the design of the study, assessed all journals quality using the same holistic rating scale. Two research assistants assessed each journal independently, following procedures similar to those used to assess the compositions. The Kappa value obtained was .84, considered as very good according to Landis and Koch [56].

#### Prior achievement

Prior achievement in Portuguese language was obtained from students’ writing quality scores on three compositions written between April and June from the previous school year (third grade). Two independent research assistants scored the compositions by following the same procedures as described above. Compositions were scored on fourteen 5-point *Likert* scales (1 = low quality, 5 = highly quality), ranging from 0 to 65 points (*M* = 50.46, *SD* = 8.63). Cohen’s Kappa coefficient showed an inter-rater agreement of .87, which can be labeled as “almost perfect” according to authors [63].

### Data analyses

Considering the hierarchical nature of data, a three-level hierarchical model was conducted. To avoid the enumeration of all the possible models, a data-driven strategy for selecting the best model by computing information criteria was used.

**Fig 2:**
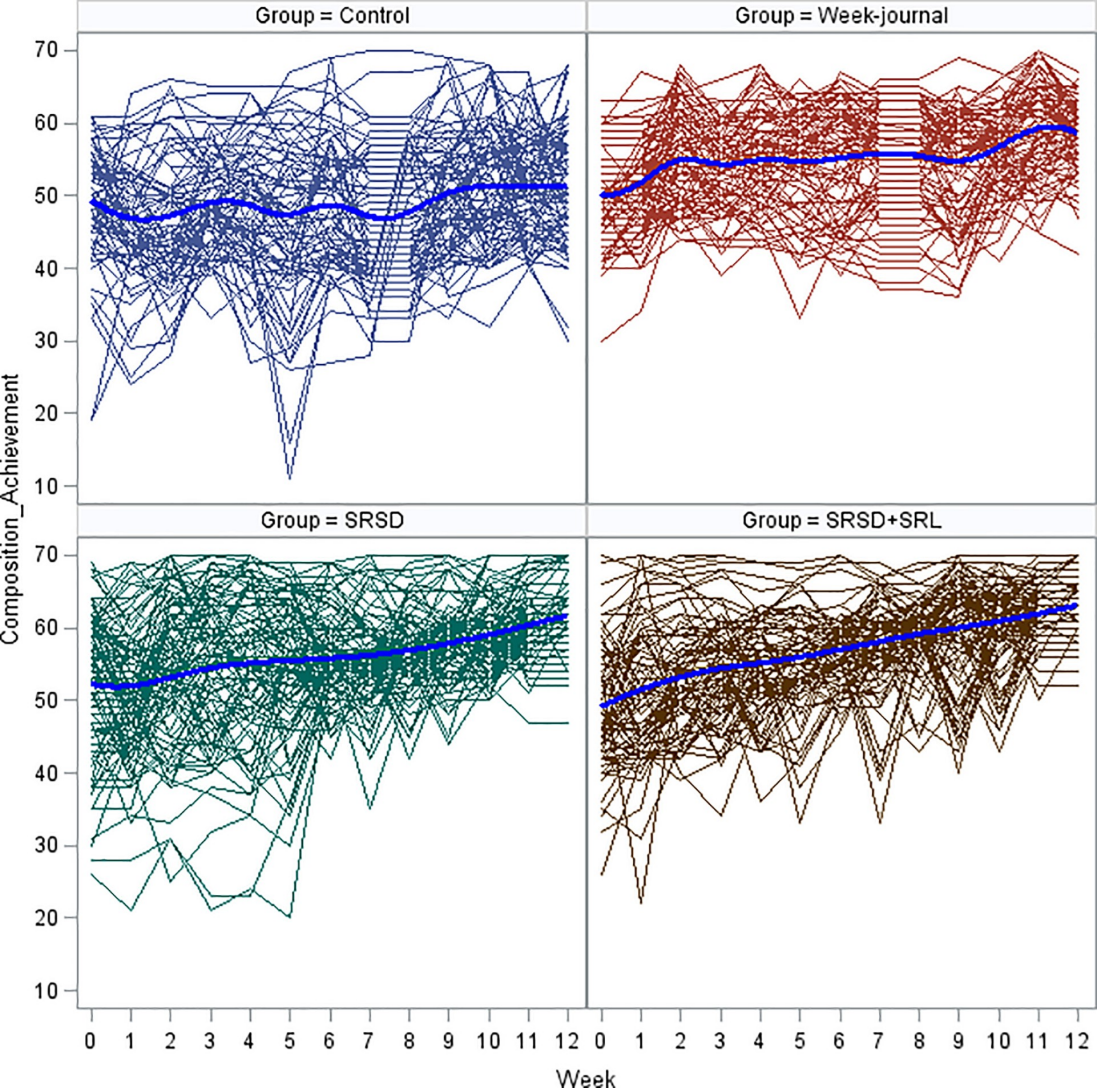
Spaghetti plot of observed data for each participant during the period under study and means (solid line) of the different treatment groups.

Fig 2 presents a “spaghetti plot” of the compositions scores (CS) by time. This plot indicates that students who received any form of treatment have increased the CS scores, although clearly there is considerable individual heterogeneity (i.e., some participants show accelerating positive trends, while others have decelerating negative trends). Some participants even have significant swings upward or downward across time of their CS response. In contrast, the trend lines appear to be approximately linear for most participants. With regard to the population level, Fig 1 shows interesting differences for the four groups across time. The group B (i.e., Week-journal) began with a moderate upturn in CS followed by a very slow increase, whereas the groups C and D (i.e., SRSD and SRSD+SRL) showed a moderate but steady and gradually accelerating upward trend up at the end of the study. The participants in the comparison group did not show an upward trend.

Visual examination suggests that the relationship displayed in Fig 2 may be nonlinear at the individual level, hence it is assumed, subject to verification, a quadratic model to describe individual change across time. To begin, the *CS* outcome at time *t* for student *i* in class *j* is modeled at level 1 by

$$\begin{array}{c} CS_{tij}=\pi_{0ij}+\pi_{1ij}\left(TIME_{tij}-L\right)+\pi_{2ij}{\left(TIME_{tij}-L\right)}^{2}+\pi_{3ij}SE\_W_{tij}+\\ \pi_{4ij}SR\_W_{tij}+\pi_{5ij}AT\_W_{tij}+\pi_{6ij}SR\_W_{tij}\times \left(TIME_{tij}-L\right)+\\ \pi_{7ij}SE\_W_{tij}\times \left(TIME_{tij}-L\right)+\pi_{8ij}AT\_W_{tij}\times \left(TIME_{tij}-L\right)+e_{tij,} \end{array}$$

where *π*_0*ij*_ is the expected outcome for student *ij* at time *L* (here the centering parameter, *L*, was a priori set at 6 weeks to avoid potential collinearity problems in the quadratic trend model), *π*_1*ij*_, the parameter associated with *TIME*, represents the rate of change in the *CS* for student *ij* at time *L* (i.e. the instantaneous rate of change when *TIME*_*tij*_ = 0), *π*_2*ij*_, the parameter associated with *TIME*^2^, describes the quadratic change in the *CS* for student *ij* (i.e. captures the curvature or acceleration regardless of the choice of location for level-1 predictors), *π*_3*ij*_ is the student’s change in *CS* due to self-efficacy in writing (*SE_W*), *π*_4*ij*_ is change in *CS* due to self-regulation in writing (*SR_W*), *π*_5*ij*_ is change in *CS* due to attitude toward writing (*AT_W*), *π*_6*ij*_ is change in *CS* due to cross-product between *SR_W* and *TIME*, *π*_7*ij*_ is change in *CS* due to cross-product between *SE_W* and *TIME*, *π*_8*ij*_ is change in *CS* due to cross-product between *AT_W* and *TIME*, and *e*_*tij*_ represents a residual.

Data from a preliminary analysis suggested considerable random variation, intercept and slope at both levels 2 and 3. The results also indicated the need to retain the main effects of time-varying predictors (i.e., *SE_W*, *SR_W* and *AT_W*) and the interaction between *SR_W* and linear *TIME* in the level-1 model but treat them as fixed instead of allowing them to change randomly across level-2 and at level-3 units. To correctly interpret the model parameters, it is important to note that all time-varying predictors were included in the model centered at its mean.

At level-2, individual differences in the random coefficients from level 1 (i.e., *π*_0*ij*_, *π*_1*ij*_, *π*_2*ij*_) were modeled as a function of student’s gender (girl = 1, boy = 0; *GEN*), prior achievement (ranging from 1 = low quality to 5 = high quality; *P_ACHIEV*), and baseline age in years (*AGE*). The *P_ACHIEV* predictor was entered into the model centered at its mean. Specifically, the following level-2 model was formulated

$$\begin{array}{l} \pi_{0ij}=\beta_{00j}+\beta_{01j}GEN_{ij}+\beta_{02j}P\_ACHIEV_{ij}+\beta_{03j}AGE_{ij}+r_{0ij},\\ \pi_{1ij}=\beta_{10j}+r_{1ij},\\ \pi_{2ij}=\beta_{20j}+r_{2ij},\\ \pi_{3ij}=\beta_{30j}{,}_{}\pi_{4ij}=\beta_{40j}{,}_{}\pi_{5ij}=\beta_{50j}{,}_{}\pi_{6ij}=\beta_{60j}, \end{array}$$

where, *β*_00*j*_ represents the average *CS* level within class *j* at time *L* (i.e. at week 6), *β*_01*j*_ indicates whether boys and girls differ in their *CS* average within class *j* after controlling for prior achievement and baseline age, *β*_02*j*_ represents the differentiating effect of prior achievement in the *CS* average within class *j* after controlling for gender and age at baseline, and *β*_03*j*_ represents the differentiating effect of age in the *CS* average within class *j* after controlling for gender and prior achievement. In addition, *r*_0*ij*_ indicates whether students nested within class *j* differed in their expected outcome at time *L*, *r*_1*ij*_ indicate whether students nested within class *j* differed significantly in their rate of change at time *L*, *r*_2*ij*_ indicates whether students nested within class *j* differed significantly in their rate of deceleration. Note that the interpretation of the quadratic coefficient does not depend on centering for time. The results suggested the need to retain the main effects of time-invariant predictors *GEN* and *P_ACHIEV* in the level-2 model, but treat them as fixed rather than allowing them to randomly vary across level-3 clusters.

Next, we explored whether students nested within classes receiving training for *CS* during 12 weeks began at a different level, or progressed over time at a different rate of growth and acceleration, than those who did not receive training. Thus, the level-3 model incorporated the treatment conditions, the explanatory variable of major interest in the current research. As previously mentioned, the 20 classes were randomized in groups of five for each of the treatment conditions: control, week-journal (*WJ*), self-regulated strategy development (*SRSD*), or *SRSD+SRL* condition. In the analysis, these four groups were compared using Helmert contrasts. Specifically, the contrast coefficients for the three group-related Helmert contrasts were: *H*_1_ = c (-1, 1/3, 1/3, 1/3), *H*_2_ = c (0, -1, 1/2, 1/2), and *H*_3_ = c (0, 0, -1, 1). The first Helmert contrast involves a comparison of subjects randomized to control versus some form of treatment. The second Helmert contrast implies to compare subjects randomized to *WJ* versus some form of *SRL*, while the goal of the third Helmert contrast is to compare the subjects randomized to *SRSD* versus *SRSD + SRL*.

This model is defined by

$$\begin{array}{l} \beta_{00j}=\gamma_{000}+\gamma_{001}H_{1j}+\gamma_{002}H_{2j}+\gamma_{003}H_{3j}+u_{00j},\\ \beta_{10j}=\gamma_{100}+\gamma_{101}H_{1j}+\gamma_{102}H_{2j}+\gamma_{103}H_{3j}+u_{10j},\\ \beta_{20j}=\gamma_{200}+\gamma_{201}H_{1j}+\gamma_{202}H_{2j}+\gamma_{203}H_{3j}+u_{20j},\\ \beta_{30j}=\gamma_{300},\;\beta_{40j}=\gamma_{400},\;\beta_{50j}=\gamma_{500},\;\beta_{60j}=\gamma_{600},\\ \beta_{01j}=\gamma_{010},\;\beta_{02j}=\gamma_{020}, \end{array}$$

where *γ*_000_ is the overall mean intercept in the four treatment conditions at time *L*, *γ*_001_ is the difference between the control and treatment groups in the mean response at time *L*, *γ*_002_ is the difference between the *WJ* and some form of *SRL* groups in the mean response at time *L*, *γ*_003_ is the difference between the *SRSD* and *SRSD+SRL* groups in the mean response at time *L*, *γ*_100_ is the mean slope, or rate of change in the mean response over time in four treatment conditions, *γ*_101_ is the difference between the control and treatment groups in the rate of change in the mean response over time, *γ*_102_ is the difference between the *WJ* and some form of *SRL* groups in the rate of change in the mean response over time, *γ*_103_ is the difference between the *SRSD* and *SRSD+SRL* groups in the rate of change in the mean response over time, *γ*_200_ is the rate of acceleration in the mean response over time in the four treatment conditions (a measure of the upward or downward curve), *γ*_201_ is the difference between the control and treatment groups in the rate of acceleration in the mean response over time, *γ*_202_ is the difference between the *WJ* and some form of *SRL* groups in the rate of acceleration in the mean response over time, is the difference between the *SRSD* and *SRSD+SRL* groups in the rate of acceleration in the mean response over time, and *u*_00*j*_, *u*_10*j*_ and *u*_20*j*_ are the level 3 residuals allowing class *j*’s subjects to deviate from population averages.

By substitution, a single regression equation for the three-level growth model is given by

$$\begin{array}{l} CS_{tij}=\gamma_{000}+\gamma_{001}H_{1j}+\gamma_{002}H_{2j}+\gamma_{003}H_{3j}+\gamma_{010}GEN_{ij}+\gamma_{020}P\_ACHIEV_{ij}+\\ \;\gamma_{100}\left(TIME_{tij}-L\right)+\gamma_{200}{\left(TIME_{tij}-L\right)}^{2}+\gamma_{300}SE\_W_{tij}+\gamma_{400}SR\_W_{tij}+\\ \;\gamma_{500}AT\_W_{tij}+\gamma_{600}SR\_W_{tij}\times \left(TIME_{tij}-L\right)+\gamma_{101}H_{1j}\times \left(TIME_{tij}-L\right)+\\ \;\gamma_{102}H_{2j}\times \left(TIME_{tij}-L\right)+\gamma_{103}H_{3j}\times \left(TIME_{tij}-L\right)+\gamma_{201}H_{1j}\times {\left(TIME_{tij}-L\right)}^{2}+\\ \;\gamma_{202}H_{2j}\times {\left(TIME_{tij}-L\right)}^{2}+\gamma_{203}H_{3j}\times {\left(TIME_{tij}-L\right)}^{2}+u_{10j}\left(TIME_{tij}-L\right)+\\ \;r_{1ij}\left(TIME_{tij}-L\right)+u_{10j}{\left(TIME_{tij}-L\right)}^{2}+r_{1ij}{\left(TIME_{tij}-L\right)}^{2}+u_{00j}+r_{0ij}+e_{tij} \end{array}$$

which illustrates that the *CS* may be viewed as a function of the overall intercept (*γ*_000_), the effect of the comparison *H*_1_(*γ*_001_), the effect of the comparison *H*_2_(*γ*_002_), the effect of the comparison *H*_3_(*γ*_003_), the effect of student’s *GEN*(*γ*_010_), the effect of student’s *P*_*ACHIEV*(*γ*_020_), the linear effect of *TIME*(*γ*_100_), the quadratic effect of *TIME*(*γ*_200_), the effect of self-efficacy in writing *SE*_*W*(*γ*_300_) the effect of regulation in writing *SR*_*W*(*γ*_400_) the effect of attitude toward writing *AT*_*W*(*γ*_500_) and the interaction effects, *SR*_*W* by *TIME*(*γ*_600_), *H*_1_ by *TIME*(*γ*_101_), *H*_2_ by *TIME*(*γ*_102_), *H*_3_ by *TIME*(*γ*_103_), *H*_1_ by *TIME*^2^(*γ*_201_), *H*_2_ by *TIME*^2^(*γ*_202_), and *H*_3_ by *TIME*^2^(*γ*_203_), plus a random error: $\left(u_{10j}+r_{1ij}\right)\times TIME_{tij}+\left(u_{20j}+r_{2ij}\right)\times TIME_{tij}^{2}+u_{00j}+r_{0ij}+e_{tij}$. The variables *u*_00*j*_, *u*_10*j*_ and *u*_20*j*_ are random class effects associated with intercept, linear time slope, and quadratic time slope, respectively; *u*_0*ij*_, *u*_1*ij*_ and *u*_2*ij*_ are random effects for clustering of students within classes associated with intercept, linear time slope, and quadratic time slope, respectively; and *e*_*tij*_ represents a residual.

Consistent with common practice in multilevel modeling, we assume that the random effects associated with classes are independent of the random effects associated with students nested within classes, and that all random effects are independent of the level 1 random components. It is also assumed that the residuals are normally distributed with zero means and uncorrelated with respective right-hand covariates. Multilevel analysis was conducted by fitting a variance components structure with parameters estimated by the full maximum likelihood (ML) estimation as implemented in PROC MIXED of [64].

## Results

### Descriptive analyses

Prior to conducting the analysis, the distribution of the data of the different samples for the outcome variable (composition skills–*CS_W*) and time-dependent covariates (i.e., *SE_W*, *SR_W* and *AT_W*) were examined. The extent of variations of skewness and kurtosis for the variables were included in the model, as well as the means and standard-deviations presented in Table 1. As shown in this table, the skewness values are generally within the range (i.e., ± 1) of what is considered a reasonable approximation to the normal curve. Looking at the kurtosis, it is necessary to note that depending on the time of the measurements, the variables are very slightly platykurtic (i.e., its peak is just a bit shallower than the peak of a normal distribution) or very slightly leptokurtic (i.e., its central peak is just a bit higher than the peak of a normal distribution). As a result, it can be concluded that the values for skewness and kurtosis remain within allowable limits for all the time periods.

**Table 1:** Descriptive statistics of written composition skills and time-varying covariates across time.

Week													
CS_W	0	1	2	3	4	5	6	7	8	9	10	11	12
*N*	364	363	366	366	362	366	365	366	365	362	365	366	364
Mean	50.47	50.07	52.47	53.01	53.62	52.94	54.61	53.98	54.95	55.83	56.78	58.38	58.66
*SD*	8.35	8.44	8.91	8.04	8.47	9.32	7.41	8.37	7.80	7.95	7.02	6.89	7.09
*SK*	-.45	-.16	-.18	-.28	-.46	-.99	-.42	-.65	-.81	-.43	-.57	-.55	-.81
*KUR*	.67	.49	-.39	.36	.34	.58	.01	.02	.10	-.40	.02	-.16	.85
Week													
SE_W	0	1	2	3	4	5	6	7	8	9	10	11	12
*N*	364	363	366	366	362	366	365	366	365	362	365	366	364
Mean	2.30	2.27	2.29	2.31	2.36	2.41	2.45	2.57	2.59	2.73	2.79	2.88	2.97
*SD*	.43	.43	.42	.51	.47	.45	.42	.52	.59	.65	.68	.71	.71
*SK*	.11	.05	.27	.29	.28	-.24	-.12	.28	.19	.11	.21	-.02	-.22
*KUR*	.58	.39	1.54	.55	1.13	-.06	.62	.06	-.37	-.54	-.70	-.99	-.98
Week													
SR_W	0	1	2	3	4	5	6	7	8	9	10	11	12
*N*	364	363	366	366	362	366	365	366	365	362	365	366	364
Mean	3.82	3.96	4.07	4.20	4.26	4.25	4.27	4.30	4.29	4.31	4.30	4.29	4.31
*SD*	.61	.67	.68	.67	.69	.73	.64	.70	.75	.69	.71	.72	.72
*SK*	-.24	-.81	-.87	-.93	-1.02	-.96	-1.10	-.89	-1.11	-1.06	-1.09	-.91	-.86
*KUR*	-.44	.44	.61	.41	.82	.34	.88	.58	1.11	.82	-1.09	.24	.05
Week													
AT_W	0	1	2	3	4	5	6	7	8	9	10	11	12
*N*	364	363	366	366	362	366	365	366	365	362	365	366	364
Mean	2.77	2.77	2.86	2.90	2.90	2.99	3.02	3.02	3.10	3.10	3.15	3.13	3.18
*SD*	.59	.62	.60	.63	.61	.63	.60	.64	.58	.61	.62	.64	.68
*SK*	-.24	-.23	-.41	-.52	-.38	-.45	-.49	-.65	-.61	-.56	-.60	-.63	-.76
*KUR*	-.44	-.39	.03	.05	-.17	-.33	.18	.00	-.08	-.15	-.10	-.09	-.05

Note. N = sample size; SD = Standard deviation; SK = Skewness; KUR = Kurtosis; CS_W = Written composition skills per week; SE_W = Self-efficacy in writing per week; SR_W = Self-regulation in writing per week; AT_W = Attitude toward writing per week.

### Multilevel analyses

#### Selecting the best model

To address the goals of the present study (i.e. compare the performance of subjects receiving training in writing skills with the performance of subjects with no training, verify whether all treatments have the same effectiveness, and determine which of two treatments (C or D) was more effective); first the best linear mixed model to the *CS* use data was selected. Tables 2 and 3 present the results of fitting eight growth curve models to the *CS* data using full ML in SAS PROC MIXED. Table 2 summarize the results for five multilevel models applied to *CS* data as follows: the unconditional two-level growth model (A) examined the standard linear change, the unconditional two-level growth model (B) and three-level growth model (C) examined the quadratic change, the conditional three-level growth model (D) examined the effects of the time-varying predictors and their interactions through time, and the conditional three-level growth model (E) examined the process of adding time-invariant predictors to models. Table 3 presents the models that incorporate the effects of treatment conditions, both with and without the heterogeneous variance specifications at level 1.

**Table 2:** Results of fitting alternative multilevel models for change to the composition skills data.

	Model A		Model B		Model C		Model D		Model E	
Fixed Effect	Estimate	SE	Estimate	SE	Estimate	SE	Estimate	SE	Estimate	SE
*Mean*	54.289***	.307	54.099***	.364	54.139***	.955	54.081***	.951	55.442***	1.179
*TIME*	.657***	.040	.657***	.040	.640***	.105	.559**	.098	.551***	.096
*TIME*^*2*^			.014	.008	.018	.019	.019	.019	.019	.018
*SE_W*							.466*	.193	.464*	.191
*SR_W*							.727***	.182	.642***	.177
*AT_W*							.611**	.198	.531**	.193
*SE_W × TIME*							.036	.050	.037	.050
*SR_W× TIME*							-.093*	.042	-.121**	.042
*AT_W× TIME*							-.005	.047	.012	.047
*AGE*									.064	.332
*GEN*									.937**	.342
*P_ACHIEV*									3.160***	.217
Random Effect	Estimate	SE	Estimate	SE	Estimate	SE	Estimate	SE	Estimate	SE
Level-1 (within-subject variance)										
Random error	25.951***	0.579	23.286***	0.564	23.288***	0.564	23.252***	0.545	23.146***	0.543
Level-2 (between students within classes variances)										
6-week status	32.560***	2.555	44.346***	3.581	27.644***	2.413	25.740***	2.283	16.300***	1.573
Linear rate	.453***	.044	.467***	.044	.277***	.031	.257***	.029	0.247***	0.029
Quadratic rate			.015***	.002	.008***	.002	.008***	.002	0.008***	0.002
Level-3 (between-classes variances)										
6-week status					16.468**	5.759	16.415**	5.712	17.122**	5.772
Linear rate					.199**	.070	.165**	.059	.160**	.058
Quadratic rate					.006**	.002	.005**	.002	.005**	.002
Goodness-of-fit										
Deviance	30516.5		30326.7		30011.4		29960.6		29441.1	
AIC	30528.5		30346.7		30043.4		30004.6		29495.1	
BIC	30552.0		30385.7		30059.3		30026.6		29516.0	

Note:

*p < .05

**p < .01

***p < .001

**Table 3:** Results of fitting alternative homogeneous and heterogeneous level-1 variance models for change to the composition skills data.

	Model F		Model G				Model H			
Fixed Effect	Estimate	SE	Estimate	SE	DF	*|t|*	Estimate	SE	DF	*|t|*
*Intercept*, ${\hat{\gamma }}_{000}$	53.672***	.970	53.670***	.590	16	90.90	53.710***	.593	16	90.56
*TIME*, ${\hat{\gamma }}_{100}$	.591***	.096	.553***	.088	4670	6.28	.552***	.088	4670	6.26
*TIME*^*2*^, ${\hat{\gamma }}_{200}$	.021	.018	.022	.018	4670	1.21	.022	.018	4670	1.26
*SE_W*, ${\hat{\gamma }}_{300}$	.494*	.186	.469*	.187	4670	2.51	.390*	.183	4670	2.14
*SR_W*, ${\hat{\gamma }}_{400}$	.639***	.177	.647***	.179	4670	3.65	.660***	.178	4670	3.72
*AT_W*, ${\hat{\gamma }}_{500}$	.553**	.192	.517**	.192	4670	2.69	.604**	.190	4670	3.17
*SR_W×TIME*, ${\hat{\gamma }}_{600}$	-.116**	.040	-.120**	.040	4670	2.96	-.137***	.039	4670	3.48
*GEN*, ${\hat{\gamma }}_{010}$	.926**	.342	.933**	.341	4670	2.74	.849*	.338	4670	2.51
*P_ACHIEV*, ${\hat{\gamma }}_{020}$	3.154***	.216	3.155***	.217	4670	14.59	3.139***	.215	4670	14.63
*H*_*1*_, ${\hat{\gamma }}_{001}$			5.168***	.799	16	6.47	5.165***	.803	16	6.44
*H*_*2*_, ${\hat{\gamma }}_{002}$			1.695**	.587	16	2.89	1.738**	.579	16	3.01
*H*_*3*_, ${\hat{\gamma }}_{003}$			.716	.508	16	1.42	.709	.506	16	1.40
*H*_*1*_ *× TIME*, ${\hat{\gamma }}_{004}$			.274	.149	4670	1.83	.272	.150	4670	1.81
Random Effect	Estimate	SE	Estimate	SE			Estimate	SE		
Homogeneous Level-1 variance (within-subject)										
Random error, ${\hat{\sigma }}^{2}$	23.159***	0.545	23.158***	0.545						
Heterogeneous Level-1 variances (within-subject)										
Random error (Control), ${\hat{\sigma }}_{1}^{2}$							29.994***	1.333		
Random error (WJ), ${\hat{\sigma }}_{2}^{2}$							14.270***	.664		
Random error (SRSD), ${\hat{\sigma }}_{3}^{2}$							27.159***	1.259		
Random err (SRSD/SRL), ${\hat{\sigma }}_{4}^{2}$							22.714***	1.049		
Level-2 (between students within classes variances)										
*L*-status, ${\hat{\tau }}_{\pi 00}$	16.294***	1.573	16.308***	1.576			15.914***	1.554		
Linear rate, ${\hat{\tau }}_{\pi 11}$	0.246***	0.029	.245***	.029			.222***	.028		
Quad rate, ${\hat{\tau }}_{\pi 22}$	0.008***	0.002	.008***	.002			.007***	.002		
Level-3 (between-classes variances)										
*L-*status, ${\hat{\tau }}_{\beta 00}$	17.113**	5.768	5.261**	2.108			5.356**	2.122		
Linear rate, ${\hat{\tau }}_{\beta 11}$	.160**	.058	.131**	.048			.133**	.048		
Quad rate, ${\hat{\tau }}_{\beta 2}$	.005**	.002	.005**	.002			.005**	.002		
Goodness-of-fit										
Deviance	29441.8		29407.5				29275.4			
AIC	29485.8		29459.5				29333.4			
BIC	29507.7		29485.3				29362.3			
Intraclass correlation (ICC) and design effects (DEFT)										
Level	ICC	SE		95% Asymptotic Confidence Interval					DEFT	
Class	0.1133	0.0226		0.0689 0.1577					1.7274	
Student | Class	0.4907	0.0192		0.4529 0.5284					2.6246	

*Note*:

*p < .05

**p < .01

***p < .001

To facilitate the selection of the best model, results (not shown in the table due to space) corresponding to the unconditional means model (i.e., a no-change trajectory model) were described. The estimated outcome grand mean across all occasions and students was 54.29 (*p* < .001), which suggests that between the first and the twelfth week, the average *CS* is non-zero. Examining the variance components, we found statistically significant variability both within-students (31.55, *p* < .001) and between-students (39.37, *p* < .001). Findings allowed to conclude that *CS* outcome varies from week to week, and also that students differ from each other.

To determine whether the unconditional means model was preferable to Model A, the compound null hypothesis was tested on a set of differences between models (e.g., regarding the linear growth rate, its associated variance components and covariance between slope and intercept—this last term is not shown in the table due to space). The difference in deviance statistics, (31830.5–30516.5) = 1314, far exceeds 16.27, the 0.001 critical value of a χ^2^ distribution on 3 degrees freedom (*df*), allowing to reject the null hypothesis (*H*_*0*_) at the *p* < .001 level stating that all the three parameters are simultaneously 0. Hence, the unconditional two-level growth model provides a better fit than the unconditional means model. It is possible to conclude that Model A is the best fit model? Comparison of Models B and A suggest a positive response. Comparing deviance statistics for pair of nested models yields a difference of 189.8. As this exceeds the .001 critical value of a χ^2^ distribution on 4 *df* (18.46), the *H*_*0*_ is rejected, and we may conclude that there is potentially predictable variation in the acceleration rate across students. For Model B, despite the variance for quadratic component of change (*r*_2*i*_) being statistically significant (*p* < .001), its associated fixed effect (*TIME*^2^) is not. The tests associated with the random acceleration parameter indicate that there is substantial variation in the quadratic rates across students. The test for the fixed effect indicates that the average value of these rates is indistinguishable from 0. Thus, the trend across time is essentially linear at the population level but curvilinear at the individual level.

Then the unconditional quadratic three-level Model C was compared to the unconditional quadratic two-level Model B. Since students are nested within classes, and may vary considerably among themselves, a three-level model of level-1 occasions nested within level-2 students nested within level-3 classes was also used to analyze this clustered longitudinal design. As there are only 20 classes, *CS* dataset is not ideal for building a three-level growth model, but it can still be useful for descriptive purposes. As indicated in Table 2, the deviance statistics and number of estimated parameters for the unconditional Model C were 30011.4 and 16, respectively. The likelihood ratio test comparing the Model C to Model B yields a deviance difference statistically significant at any alpha level we might reasonably select (30326.7–30011.4 = 315.3, with 6 *df*, p < .001). Findings indicate that the more complex model provides the better fit. Each information criterion is consistent with that judgment.

Because we are interested in finding a level-1 individual growth model that describes the fundamental structure of these data, additional time-varying predictors and interactions among level-1 predictors and *TIME* (i.e., *SE_W*, *SR_W*, *AT_W*, *SE_W* × *TIME*, *SR_W* × *TIME*, and *AT_W* × *TIME*), but also the required additional variance and covariance components (see Model D) were included. Although not shown in the Table 2, the covariance components were not constrained to be 0. When comparing the Model D with the Model C, there is significant evidence that the model incorporating the main effects of time-dependent covariates and interactions fits better (i.e. the difference in deviances was (30011.4–29960.6) = 50.8; *df* = 6, *p* < 0.001). Having identified an appropriate level-1 model, the additional effects of time-invariant predictors were included in the level-2 model (i.e., *AGE*, *GEN* and *P_ACHIEV*). For Model E (i.e., model that incorporates time-varying predictors, time-invariant predictors, and the level-1 interactions), the deviance statistic was 29441.1 with 25 *df*, and 29960.6 with 22 *df* for Model D (i.e. model that only incorporates time-varying predictors and the level-1 interactions). As a result, the likelihood ratio test statistic was 518.5 with 3 *df* (*p* < .001), which provides strong evidence for Model E. Although the Model E provides a more realistic representation of the pattern of change in *CS* scores than Model D, the Model E contain terms that are not necessarily required. In this paper an even more parsimonious model will be assessed (i.e. Model F). Model F is a simplification of Model E in which the main effect of *AGE* and non-significant level-1 interaction terms are removed. Comparing the last two models each other, we find a trivial difference in deviance of 0.7 on 3 *df*, showing that the elimination of *AGE*, *SE_W* by *TIME* and *AT_W* by *TIME* has hardly changed the goodness of fit.

After running the appropriate model selection at level-2 for the *CS* use data, we examined the performance of subjects receiving training in writing skills with the performance of subjects who did not receive such training, and the performance of participants receiving treatment in different modalities. Model G in Table 3 presents the results of fitting this model to data. The final conditional model (Model G) included three class-level variables (i.e., the aforementioned set of Helmert contrasts for group), two student-level variables (*GEN* and *P_ACHIEV*) and five within level-1 repeated observations (*TIME*, *TIME*^*2*^, *SE_W*, *SR_W* and *AT_W*). The cross-product between *SR_W* and *TIME* and cross-level interaction term *H*_1_ by linear *TIME* (i.e., difference between the control and treatment groups across time) were also included in the Model G. Data in Table 3 and indicated that adding the three group-related Helmert contrasts (i.e., *H*_1_, *H*_2_ and *H*_3_) cross-level interaction between *H*_1_ and *TIME* to the model which decreased the deviance from 29441.8 to 29407.5, a decrease of 34.3. This change in deviance is tested at 4 *df* using the χ^2^ statistic and was found to be significant.

It might appear, that Model G is preferable. But before proceeding to examine Model G in depth, we considered the possibility that the residual variances at level 1 may depend on treatment groups (see, [56]). Returning to Fig 1, we note that participants display considerable heterogeneity across the groups. Thus, we might hypothesize that residual variance at level 1 in these data is different for the four conditions. Table 3 presents estimates for homogeneous variances (Model G) and for heterogeneous variances that occurs at level-1 (Model H). The likelihood ratio test comparing Model G to Model H, shows that the deviance declined 132.1 (29407.5–29275.4), which far exceeds the .05 critical value of a χ^2^ distribution on 3 *df*. We therefore may reject the null hypothesis stating that all four variances are equal and conclude that the heterogeneous model fits this data better than the simple homogeneous level-1 specification. For this reason, Model H was adopted as our “final model” (see Table 3). The AIC (BIC) weight of this model (> 0.97) implies that there is a high probability that this is the best model among all of the examined models.

#### Analysis of the selected multilevel model

Once a suitable final model was selected, the results for the fixed effects corresponding to Model H were analyzed (see Table 3). The comparison of the regression coefficients allows to conclude that the constant (${\hat{\gamma }}_{000}$ = 55.408; *p* < .001) and linear trend terms (${\hat{\gamma }}_{201}$ = .552; *p* < .001) are significant. The intercept being significant is not particularly meaningful (i.e. indicates that *CS* scores are different than zero at midpoint of time). However, because the trend is essentially linear at the population level, we may conclude that participants are improving across time. On the contrary, the quadratic term is nonsignificant at the individual level (${\hat{\gamma }}_{200}$ = .022; *p* < .208). Similar inspection of the other parameter estimates in Model H shows that *CS* score was positively associated with prior achievement (${\hat{\gamma }}_{020}$ = 3.139; *p* < .001), *SE_W* (${\hat{\gamma }}_{300}$ = .390; *p* < .0027), *SR_W* (${\hat{\gamma }}_{400}$ = .660; *p* < .001) and *AT_W* (${\hat{\gamma }}_{500}$ = .604; *p* < .002). On the other hand, the *CS* score was negatively associated with the cross-product between the self-regulation and linear time (${\hat{\gamma }}_{600}$ = -.137; *p* < .001). The relationship between the self-efficacy, attitude toward writing and the *CS* score were constant across time (i.e., no time interactions with these time-varying covariates, see Model D in Table 2). We also found that the gender effect was significant (${\hat{\gamma }}_{010}$ = .847; *p* < .0012), suggesting that performance in *CS* was higher for girls than for boys.

At class level, the estimates of γ_001_, γ_002_, γ_003_, and γ_101_, and their estimated standard errors are of primary interest in Model H. Table 3 indicates that the difference between the control and treatment groups in the mean response at the midpoint of time was significantly different from zero (${\hat{\gamma }}_{001}$ = 5.165; *p* < .0001). This indicates that the intervention had a statistically significant effect on *CS* score. In addition, due to the marginally significant effect of cross-level interaction between *H*_1_ and linear *TIME* (${\hat{\gamma }}_{101}$ = .272; *p* < .068), it seems that this benefit increases over time. The regression coefficients of the *H*_2_ contrast were inspected to determine whether a differential performance in the mean response *CS* had occurred in the intervention *WJ* group in comparison with groups C and D. In Model H the effect of γ_002_ was estimated to be 1.738 while its corresponding standard error was estimated to be 0.579. The ratio was 3.01 and the *p*-value was approximately 0.008, which indicates a significant benefit for participants who received treatments C and D (i.e., *SRSD* or *SRSD* + *SRL*) in relation to participants of *WJ* group at the midpoint, and further suggests that this effect does not vary significantly across time (i.e., no time interactions with the second and third Helmert contrasts). Finally, regarding *H*_*3*_, although performance is higher for group *SRSD + SRL*, no evidence found differences in *CS* scores among *SRSD* and *SRSD* + *SRL* intervention conditions (${\hat{\gamma }}_{003}$ = .709; *p* < .179).

Finally, following the procedure of Vallejo and colleagues [65] in examining statistical power to detect a significant group-by-time-interaction (i.e., H1 × TIME), a power below the often-mentioned benchmark of 0.80 was obtained; specifically, the *post hoc* power was found to be approximately 0.44.

Before describing the structure of the random-effects model matrix, we included two intraclass correlations (ICCs) for this three-level hierarchical model (see Table 3, bottom panel). The first is the level-3 ICC at the class level, the correlation among quality of compositions from different second level students nested on the same class. The second is the level-2 ICC at the student-within-class level, the correlation among quality of compositions measured at different time points in the same student and class. We found that the quality of compositions is strongly correlated within the same student and class, but only slightly correlated within the same class, while this ICC is non-negligible. Table 3 (bottom right panel) also displays the design effect (DEFT) indices at levels 2 and 3 in Table 3. DEFT is used to determine how much larger the standard errors estimates will be (considering clustering compared to the analysis that ignore clustering). For example, for the ICC in level two (class) (see Table 3) the unconditional DEFT is expected to be 1.73; meaning that standard errors would only capture a little more than one-half of the true sampling variability if the third-level was ignored.

Analyzing the variances estimates, data shows that at the student-level the estimate constant variance $\left({\hat{\tau }}_{\pi 00}\right)$ is much larger than the estimate linear trend component $\left({\hat{\tau }}_{\pi 11}\right)$, which is much larger than the estimated quadratic trend component $\left({\hat{\tau }}_{\pi 22}\right)$. In terms of relative percentages, these three represent 98.5, 1.4, and 0.1, respectively, of the sum of the estimated individual variance terms. A similar result was observed at class level (${\hat{\tau }}_{\beta 00}$, ${\hat{\tau }}_{\beta 11}$ and ${\hat{\tau }}_{\beta 22}$), although heterogeneity in trends across time becomes smaller as the order of trend terms increased. Note also that final estimation of level-1 and level-2 variance components has been affected very little by model respecification (Model F *vs*. Model G/H). However, the final estimation of level-3 variance components has been substantially diminished when compared with the parameters estimates for Model G/H.

## Discussion

In this study, the impact of three types of writing interventions (i.e., week-journals, SRSD, SRSD plus story-tool) on the quality of writing compositions was analyzed using a longitudinal cluster-randomized controlled design. Moreover, to analyze the effects of the four intervention conditions on writing composition skills, a set of covariates were controlled (i.e. self-regulation in writing, self-efficacy in writing, attitude towards writing, prior achievement in writing, gender and age). These variables have been selected due to previous findings on their positive effects on students’ writing quality.

The current research contributes to literature due to three major aspects. To the best of our knowledge this is the first study that examined the benefits of a free writing activity (i.e., week-journals) in comparison with two other instructional writing programs. Moreover, this study contributes to literature by adding a story-tool that enhances self-regulation to the SRSD model. Lastly, this study analyzes the effects of three types of writing intervention by conducting a longitudinal cluster-randomized controlled design using a multilevel modeling analysis. This complex design of the randomized cluster groups over time allowed for the effectiveness of this educational intervention to be measured in a real-life setting, but with robust control. Current findings are expected to provide relevant data that may help researchers and educators improve their work to increase the students’ quality of writing.

### The effectiveness of writing interventions on writing quality

Findings support our hypothesis by showing differences between the treatment groups in students’ writing quality over time, but with some reserves. Firstly, it was found that the students enrolled in the three intervention groups exhibited higher levels of writing quality in their composition when compared to those of students with no intervention (i.e., comparison group). These findings indicate that all writing intervention groups showed a positive and significant impact on students writing quality, which increased the intervention time. These findings are consistent with literature that reports the benefits of writing journals [15], of participating in instructional writing programs as SRSD (e.g., [3,5,30,60–61,66]), and of participating in general SRL training programs using story-tools [37–41]. Moreover, it was observed that the evolution of the writing quality of the three intervention groups was, overall, essentially linear and positive, indicating a constant acquisition of writing skills occurring over time.

Secondly, it was found that students who participated in the instructional programs (i.e., SRSD and SRSD plus story-tool) exhibited higher writing quality than students who wrote week-journals. Furthermore, Fig 2 also shows that the writing quality of students in the week-journals condition achieved a plateau after three weeks, while the writing quality of students in the two instructional programs continue to improve after that period. These findings are consistent with those of colleagues [15] showing that in order to master higher levels of writing skills and overcome the plateau effect it would be necessary to enroll in instructional writing programs designed to promote writing quality. These results are also consistent with data from the meta-analysis by Graham et al. [5], which found that studies involving strategy instruction using the SRSD model produced a statistically positive effect on students’ writing quality with an effect size (ES) of 1.17 in average. On the other hand, investigations enrolling students in free writing activities (e.g., writing about a free topic) produced an average weighted ES of 0.30 [5].

Finally, one important goal of this research was to learn the impact of adding the usage of story-tools to SRSD intervention on the writing quality. Students’ participating in SRSD plus story-tool instruction showed a higher writing quality than their peers in the SRSD condition; however, the differences found were not statistically significant. This finding may be due to the fact that the SRSD model includes self-regulation strategies focused on writing of compositions (e.g., goal setting, self-assessment) (e.g., [28,60,67]), and that the usage of the story-toll in classes was not focused on writing, but on the promotion of general SRL strategies. In the post-intervention evaluation meeting, teachers in the condition SRSD plus story-tool instruction enthusiastically shared their students’ scores in the composition section in the national standardized exam in Portuguese language, which counts as 30% of their overall grade. As this issue was brought to discussion, the teachers in the other conditions were invited to share the results of their students (i.e. for the comparison group, scores ranged between 5 and 30 points (*M* = 18.68, *SD* = 5.46); for the Week-journals group, scores ranged between 10 and 30 points (*M* = 19.24, *SD* = 3.88; for the SRSD group, scores ranged between 11 and 29 points (*M* = 20.35, *SD* = 4.99); and for the SRSD plus story-tool group between 12 and 30 points (*M* = 23.82, *SD* = 4.02). The percentage of students scoring lower than 15 points (negative scores) per condition was: 17%, 10%, 10% and 2%, respectively). Globally, participant teachers in conditions B, C and D were very happy with their students’ writing performance that far exceeded the National average score for compositions, and their expectations.

### The effects of the covariates in writing quality

For what concerns the covariates assessed in this study, our findings have supported the need and usefulness of accounting for every single covariate (i.e., self-regulation in writing, the self-efficacy in writing, the attitude towards writing, the prior achievements in writing, the gender and the age), as they have shown a positive impact on the writing quality at the end of the instructional program. Accordantly, as previous studies focused on writing have indicated, when students receive training in SRL strategies they are likely to produce texts with quality (e.g., [3,68–69]), to engage deeply in school tasks and show higher academic achievement [51]. Furthermore, when students’ show a positive attitude towards writing [34] and perceive themselves as self-efficacious in writing, they are likely to show signs of good writing quality and invest effort while carrying out a writing task [34–36]. Specifically, it was found that the prior achievement in writing composition seems to be the variable with more influence on writing composition skills. Nevertheless, a positive relationship between each moderate variable and the writing composition performance was observed, except between self-regulation in writing and time, which were found to have a negative impact, indicating that the levels of self-regulation tend to be less predictive of the writing composition skills throughout time. This may be explained by the fact that all groups tend to match, with time, their self-regulation skills as consequence of their engagement in this study. Finally, it was observed that the improvements achieved by girls were greater than those of boys. This supports previous research that has shown that girls present higher scores in writing quality than boys (e.g., [8,59,70]).

### Conclusions, limitations and implications

Globally, the improvement of students’ writing quality over time is related to the level of specialization of the writing intervention implemented. This is an important finding with strong implications for educational practice. For example, the week-journals writing activity can be easily implemented in classrooms by teachers without much effort, time, and resources (e.g., [15]), providing teachers with an opportunity to help their students improve their writing quality. Thus, school administrators, teachers, and parents may consider the usage of week-journals as a regular writing activity for all children as a preventive approach to writing difficulties. Data of the current study did not show statistical significant differences between results from SRSD and SRSD plus story tool condition, it would be useful to conduct further research on instructional writing interventions using story-tools. In the current study, stories didn’t help students significantly improve their writing quality when compared with their counterparts in condition C.

Furthermore, in the post-research evaluation meeting teachers in the condition C and D expressed with enthusiasm that their students improved not only in their writing but also in other content domains. As the majority of the participating teachers in condition D stated in the post-research evaluation meeting, *“students started to use PLEE for everything since planning their games in the playground or the steps to solve a difficult math problem*, *to evaluate the cake baked at home or at school”* (T_11_). Participants in the condition C and D added that they felt that their students started to enjoy learning and their motivation increased for learning to write, specially the struggling students. We believe that this is a relevant finding that stresses the importance of the training on writing strategies rather than the mode of delivery. Both interventions trained students in the use of writing strategies in context, and the interventions used examples to explore the strategies, and yielded similar results. The use of the stories may contribute to improvement of students general SRL [40], but as results indicate do not help improve students writing quality directly.

Despite the promising contributions referred, further research is needed to disclose the benefits of the usage of the story-tool in combination with writing instruction. In fact, implications derived should be taken cautiously due to the limitations of this study. The present study used self-reports to assess SRL strategies, attitude towards writing and self-efficacy in writing. However, self-reports did not capture real-time response demands of authentic learning environments [51]. For example, it is possible that these instruments did not capture the benefits and potential of the story-tool to improve writing quality. These possible explanations reinforce the need to include event measures in the research design likely to capture the processual nature of the variables being studied.

Moreover, future research could consider including variables that may help explain results (e.g., writing goals, anxiety towards writing, contextual variables [65]), and improve the sensitivity of the measures, (e.g., using on task measures to access SRL). Finally, given the insight provided by the data collected in the post-research meeting, future studies may explore in depth the complex process of learning writing strategies in combination with a story-tool, using qualitative methods to analyze students’ and teachers’ experiences during the program.

Furthermore, our findings indicated that students’ writing quality in the two instructional conditions increased throughout the end of the study. It would be relevant to conduct studies with a longer duration, and with more classes in each condition to learn about the efficacy of these programs and to promote the writing quality throughout time. Finally, consistent with extant literature, we believe that educators are expected to use the best evidence available to make informed decisions and design their classes instruction accordingly [71]. We hope current findings on the efficiency of different writing interventions may help educators contextualize this knowledge and develop the best writing program possible.

## Supporting information

S1 Appendix (DOCX)

S1 Questionnaire (DOCX)

S1 Data Base (SAV)
