# Supplementary material for: The impact of three types of writing intervention on students’ writing quality
Source: PLoS One. 2019 Jul 18;14(7):e0218099. doi: 10.1371/journal.pone.0218099 (PMC6638999; doi:10.1371/journal.pone.0218099)
Supplement: S1 Appendix — (DOCX) [file pone.0218099.s001.docx]

**Appendix**

| Assessment  /Sessions | **Sessions of the instructional programs** | |
| --- | --- | --- |
|  | **SRSD (group C)** | **SRSD + story-tool (group D)** |
| Session 1 | Stage 1 (4): Develop student’s prior knowledge on composition. General writing strategies (i.e., POW) presented are discussed with students. Negative beliefs that students could have about their writing performance are discussed and changed into positive beliefs (e.g., "I can do it, if I use the right strategy”). | Book chapters: 1-3; Students learn some features of the story and meet the learning strategy *planning* for the first time*.* |
| Session 2 |  | Book chapter: 4; Students are encourage to value the role of effort and commitment in their learning process. Some quotations of the story helped students to change their negative beliefs into positive (e.g., *“To learn more and grow wiser depends mainly on what each one does”).* |
| Session 3 | Stage 2 (4): Students revisit the general writing strategies (i.e., POW) and discuss the SRL strategies (i.e., self-instructions, goal setting, self-assessment and self-reinforcement) to be used before, during and after writing a story. | Book chapter: 5; Students analyze the steps of the problem solving process and practice the implementation of those steps using specific tasks. |
| Session 4 |  | Book chapter: 6; Students identify, define and apply the three phases of the self-regulatory process (i.e., plan, execute and evaluate) in the context of different daily and learning tasks. |
| Session 5 | Stage 3 (4): The planning, writing and assessing of compositions using a set of general and SRL strategies taught are modeled collaboratively in class. | Book chapter: 7; Students are faced with the importance of peer and collaborative work. |
| Session 6 |  | Book chapter: 8; Students are asked to describe and to reflect on how the colors identified each of the three phases of the SRL process and on the SRL strategies applied. |
| Session 7 |  | Book chapters: 9-10; Students reflect about the characters’ emotions and behaviors and identify similar daily life situations. Students learn to foresee and reflect about the consequences of their behavior in short- and long-term. |
| Session 8 | Stage 5: Strengthened students’ ability for independent planning, writing and assessing of stories by using the general and the SRL strategies. | Book chapters: 11-12; Students are faced with a well-known tale which representatively illustrates the use of the PLEE phases, the importance of self-setting goals and making the effort to accomplish such goals. |
| Session 9 |  | Book chapters: 13-14; Students are firstly asked to discuss and reflect about how the characters applied the steps of the problem solving process, and secondly to put those steps into practice independently. |
| Session 10 |  | Book chapters: 15 – 17; Students are asked to reflect about how the characters had to take responsibility for their acts and about the importance of effort and commitment to accomplish their main goal (i.e., finding their friend Yellow). |
| Session 11 | Stage 6: Independent performance | |
