# Supplementary material for: The impact of three types of writing intervention on students’ writing quality
Source: PLoS One. 2019 Jul 18;14(7):e0218099. doi: 10.1371/journal.pone.0218099 (PMC6638999; doi:10.1371/journal.pone.0218099)
Supplement: S1 Questionnaire — (DOCX) [file pone.0218099.s002.docx]

Survey n. 1 Date: ___/___/_____

|  |  |  |  |  |
| --- | --- | --- | --- | --- |
|  | *Really upset*  Muito aborrecido | *A little upset*  Um pouco aborrecido | *A little happy*  Um pouco feliz | Very happy  Muito Feliz |
| 1. *How do you feel about writing for fun at home?*   Como te sentes quando estás em casa e pensas em escrever? | 1 | 2 | 3 | 4 |
| 1. *How do you feel when you write in school during your free time?*   Como te sentes quando pensas em escrever na escola durante o teu tempo livre? | 1 | 2 | 3 | 4 |
| 1. *How do you feel when you start to write a new paper?*   Como te sentes quando pensas em começar a escrever? | 1 | 2 | 3 | 4 |
| 1. *How do you feel about writing during summer vacation?*   Como te sentes quando pensas em escrever durante as férias de verão? | 1 | 2 | 3 | 4 |
| 1. *How do you feel about writing instead of playing?*   Como te sentes quando pensas que tens de escrever em vez de poderes brincar? | 1 | 2 | 3 | 4 |
| 1. *How do feel about writing different kinds of papers?*   Como te sentes quando pensas em escrever diferentes tipos de texto? | 1 | 2 | 3 | 4 |
| 1. *How do you feel when the teacher asks you questions about what you write?*   Como te sentes quando os professores te fazem perguntas sobre o que escreveste? | 1 | 2 | 3 | 4 |
| 1. *How do you feel about writing in school?*   Como te sentes quando pensas em escrever (composições, poemas, diários, cartas, …) na escola? | 1 | 2 | 3 | 4 |
| 1. *How do you feel about spending free time writing?*   Como te sentes quando pensas em dedicar o teu tempo livre a escrever? | 1 | 2 | 3 | 4 |

|  | *Never*  Nunca | *Sometimes*  Poucas vezes | *Very often*  Muitas vezes | *Always*  Sempre |
| --- | --- | --- | --- | --- |
| 1. *When my class is asked to write, my paper is one of the best*   Quando o professor nos pede para escrever um texto, o meu é um dos melhores. | 1 | 2 | 3 | 4 |
| 1. *When writing a paper, it is hard for me to decide what goes first, second, third, and so on.*   Quando estou a escrever um texto, tenho dificuldades em perceber por onde começar: o que devo escrever em primeiro, em segundo, em terceiro… | 1 | 2 | 3 | 4 |
| 1. *When writing a paper, I have trouble finding the right words for what I want to say*   Quando escrevo um texto, tenho dificuldades em encontrar as palavras certas para dizer o que quero. | 1 | 2 | 3 | 4 |
| 1. *When I plan a paper, my plan is one of the best in the class*   Quando faço um plano/esquema do meu texto, o meu plano/esquema é um dos melhores da sala. | 1 | 2 | 3 | 4 |
| 1. *When writing a paper it is easy for me to keep thinking of things to say*   Quando escrevo um texto tenho facilidade em pôr as minhas ideias no papel. | 1 | 2 | 3 | 4 |

| **Pay attention while you answer**  Responde com atenção | *Never*  Nunca | *Rarely*  Poucas vezes | *Sometimes*  Algumas vezes | *Very often*  Muitas vezes | *Always*  *Sempre* |
| --- | --- | --- | --- | --- | --- |
| 1. *Before I start writing a composition, I think about what I want to say and what I have to do to complete it. For example, if I have to write a composition about a day on the beach, I make a list with my ideas, the vocabulary I want to use, I identify the main character, the follow ideas…*   Antes de começar a escrever uma composição, penso no que quero dizer e no que preciso fazer para a completar. Por exemplo, se tenho de fazer uma composição sobre um dia na praia, faço uma lista das ideias a desenvolver, do vocabulário a usar, identifico a personagem principal, as ideias secundárias, … | 1 | 2 | 3 | 4 | 5 |
| 1. *While I write a composition, at home or at school, I think about what I have to keep changing so that I can reach my goals. For example, while I’m writing my composition, I realise that I’m not being able to write what I want, I’m not using a good vocabulary and I won’t be able to finish it on time, I think what I have to do to improve it.*   Durante a escrita de composições, na escola ou em casa, penso no que tenho de ir mudando para conseguir alcançar os meus objetivos. Por exemplo, se durante a escrita da minha composição me apercebo que não estou a conseguir escrever o que quero, se não uso um bom vocabulário e se não consigo acabar no tempo previsto, penso no que tenho de fazer para melhorar. | 1 | 2 | 3 | 4 | 5 |
| 1. *I prepare my school bag the day before, checking if I am taking the books and materials I will need to learn and write my texts (compositions, poems, …).*   Preparo a minha pasta/mochila no dia anterior, verificando se levo para a escola os livros e os materiais necessários para poder trabalhar e escrever textos (composições, poemas,…). | 1 | 2 | 3 | 4 | 5 |
| 1. *When my teachers correct and grade my compositions, I think what I have to do to improve them. For example, if I received a lower grade because I made too many spelling mistakes, I think about it to improve.*   Quando o meu professor corrige e dá uma nota à minha composição, penso no que tenho de fazer para melhorar. Por exemplo, se tirei uma nota baixa porque tive muitos erros ortográficos, penso nisso para mudar. | 1 | 2 | 3 | 4 | 5 |
| 1. *I read carefully the corrections made on my compositions, so that I can see where I failed and what I have to do to improve.*   Leio com atenção as correções feitas nas minhas composições, para ver onde errei e saber o que tenho de mudar para melhorar. | 1 | 2 | 3 | 4 | 5 |
| 1. *When I write a composition, I follow the plan set.*   Quando escrevo uma composição, sigo o plano/esquema que fiz. | 1 | 2 | 3 | 4 | 5 |
| 1. *When asked to write a composition, it helps me to think about the grade I want to achieve.*   Quando tenho de fazer uma composição penso na nota que quero tirar e no que quero escrever e isso ajuda-me. | 1 | 2 | 3 | 4 | 5 |
| 1. *I check my grades with those I would like to achieve.*   Comparo as minhas notas com as notas que queria tirar. | 1 | 2 | 3 | 4 | 5 |
| 1. *I look for a quiet place, where I can concentrate, when I have to learn and write my compositions.*   Procuro um sítio calmo e onde esteja concentrado para poder escrever a minha composição e estudar. | 1 | 2 | 3 | 4 | 5 |
